# MUC16-mediated activation of mTOR and c-MYC reprograms pancreatic cancer metabolism

**DOI:** 10.18632/oncotarget.4078

**Published:** 2015-06-03

**Authors:** Surendra K. Shukla, Venugopal Gunda, Jaime Abrego, Dhanya Haridas, Anusha Mishra, Joshua Souchek, Nina V. Chaika, Fang Yu, Aaron R. Sasson, Audrey J. Lazenby, Surinder K. Batra, Pankaj K. Singh

**Affiliations:** ^1^ The Eppley Institute for Research in Cancer and Allied Diseases, University of Nebraska Medical Center, Omaha, Nebraska, USA; ^2^ Department of Biochemistry and Molecular Biology, University of Nebraska Medical Center, Omaha, Nebraska, USA; ^3^ Department of Biostatistics, University of Nebraska Medical Center, Omaha, Nebraska, USA; ^4^ Department of Surgery, University of Nebraska Medical Center, Omaha, Nebraska, USA; ^5^ Department of Pathology and Microbiology, University of Nebraska Medical Center, Omaha, Nebraska, USA; ^6^ Department of Genetics, Cell Biology and Anatomy, University of Nebraska Medical Center, Omaha, Nebraska, USA

**Keywords:** MUC16, metabolism, pancreatic cancer, c-MYC, metabolomics

## Abstract

MUC16, a transmembrane mucin, facilitates pancreatic adenocarcinoma progression and metastasis. In the current studies, we observed that MUC16 knockdown pancreatic cancer cells exhibit reduced glucose uptake and lactate secretion along with reduced migration and invasion potential, which can be restored by supplementing the culture media with lactate, an end product of aerobic glycolysis. MUC16 knockdown leads to inhibition of mTOR activity and reduced expression of its downstream target c-MYC, a key player in cellular growth, proliferation and metabolism. Ectopic expression of *c-MYC* in *MUC16* knockdown pancreatic cancer cells restores the altered cellular physiology. Our LC-MS/MS based metabolomics studies indicate global metabolic alterations in *MUC16* knockdown pancreatic cancer cells, as compared to the controls. Specifically, glycolytic and nucleotide metabolite pools were significantly decreased. We observed similar metabolic alterations that correlated with MUC16 expression in primary tumor tissue specimens from human pancreatic adenocarcinoma cancer patients. Overall, our results demonstrate that MUC16 plays an important role in metabolic reprogramming of pancreatic cancer cells by increasing glycolysis and enhancing motility and invasiveness.

## INTRODUCTION

Pancreatic cancer is one of the deadliest malignancies and is the fourth leading cause of cancer-related deaths in the United States [1]. Pancreatic ductal adenocarcinoma (PDAC) accounts for about 95% of pancreatic cancer cases and is considered the most aggressive type of pancreatic cancer [2]. Despite substantial progress in the understanding and therapy of pancreatic cancer, the overall 5-year survival rate remains about 5% [3]. The lethal nature of pancreatic cancer is mainly correlated with its late diagnosis, high metastatic capacity to nearby organs, and resistance to therapeutic agents [4]. Hence, there is an urgent need for a better understanding of the molecular mechanisms of pancreatic cancer pathogenesis that contribute to aggressiveness, metastasis and poor prognosis among patients.

Several oncogenes and tumor suppressors have been reported to play a role in the origin and pathogenesis of pancreatic cancer. In the course of the development of PDAC, multiple genetic, epigenetic, signaling and metabolic alterations contribute to cancer progression [5]. Mucins, a family of high molecular weight and heavily glycosylated proteins, are known to play important roles in pancreatic cancer pathogenesis. Mucin family members are involved in oncogenesis, metastasis and therapeutic resistance in pancreatic cancer [6, 7]. Among the several members of the mucin family, expression of MUC1, MUC4, and MUC5AC were found to be significantly elevated in the malignant stage of pancreatic cancer [8]. MUC1 and MUC4 have been reported to play significant roles in pancreatic cancer growth, proliferation, metastasis and drug resistance [6, 7, 9–11]. Recently, it has been shown that MUC16 expression is significantly increased in pancreatic cancer [12], but its role in the pathogenesis is not well understood.

Metabolic alterations are a hallmark of cancer [13]. Metabolic reprogramming provides nutrient influx to support the biomass need of rapidly proliferating tumor cells and also facilitate resistance to chemotherapy. Hence, aberrant metabolic features may provide novel therapeutic targets. Among several important metabolic alterations, most cancer cells exhibit enhanced aerobic glycolysis, known as the Warburg effect [14]. Enhanced glycolysis provides the carbon skeleton for biomass production, NADPH and ATP for proliferating cancer cells [15]. Increased glycolysis also leads to acidosis of the tumor microenvironment and facilitates local invasion of tumor cells [16]. Higher lactate levels in tumor cells also significantly correlate with tumor recurrence and metastasis of solid tumors [17]. Metabolic shift toward aerobic glycolysis is governed by several oncogenes and oncogenic signaling pathways [13]. Oncogenic signaling driven by K-Ras, a key regulator of pancreatic cancer pathogenesis, induces a series of metabolic alterations, including enhanced glycolysis and glutaminolysis, which significantly contribute to cancer cell growth and proliferation [18]. PDAC cells demonstrate significant upregulation of glycolytic genes in primary tumors as well as metastatic lesions [19]. Recently, we have demonstrated that another mucin family member MUC1 reprograms glycolytic metabolism in pancreatic cancer [20]. MUC1 also regulates genes involved in lipid metabolism [21]. In the present study we characterized the metabolic changes induced by MUC16 in pancreatic cancer cells and investigated the role of such metabolic alterations in modulating MUC16-induced motility and invasiveness.

## RESULTS

### *MUC16* knockdown leads to reduced glucose uptake and lactate secretion

**Figure 1:**
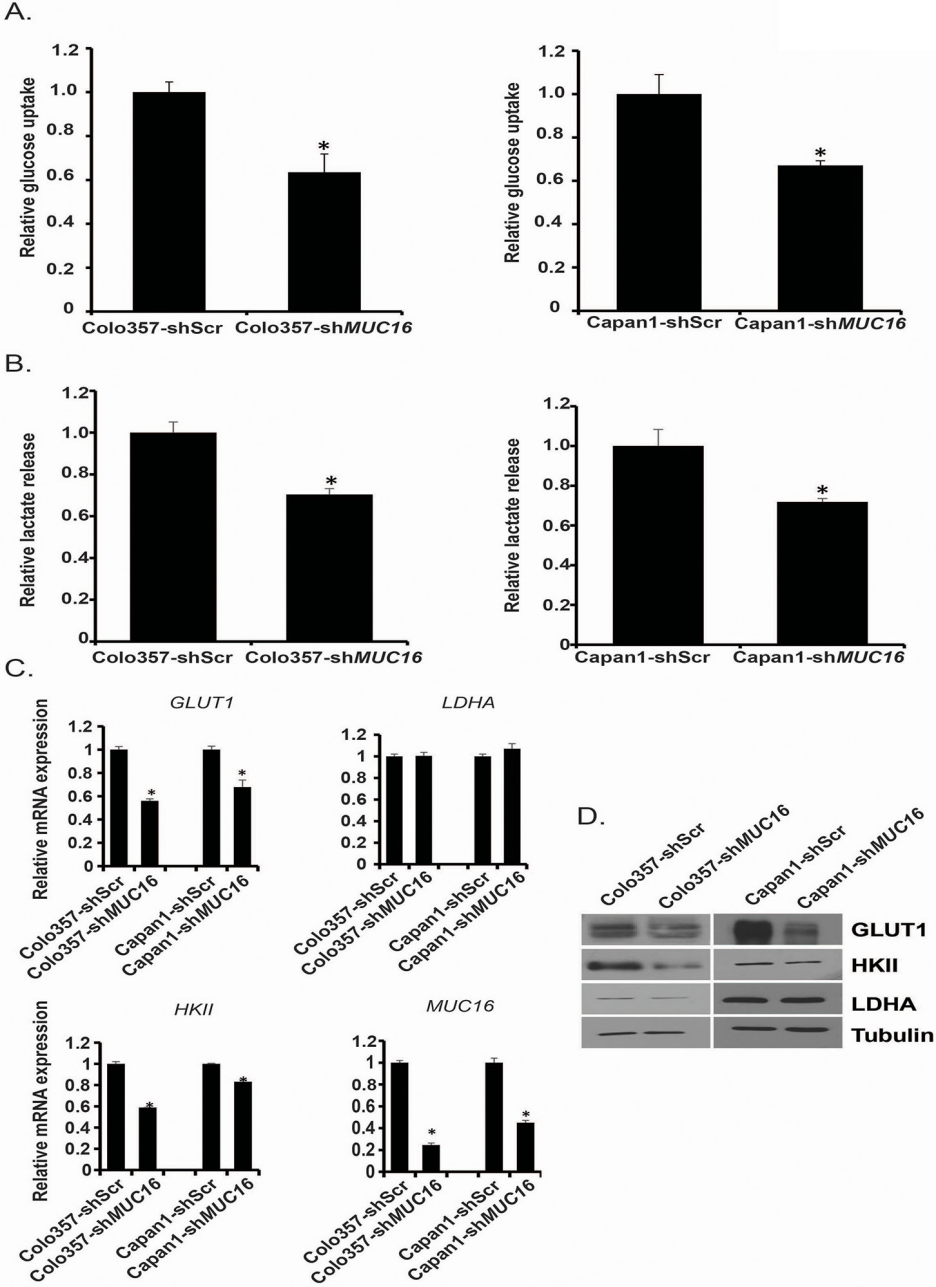
*MUC16* knockdown diminishes glycolytic activity and glycolytic gene expression **A.** Colo357-shScr, Colo357-sh*MUC16*, Capan1-shScr and Capan1-sh*MUC16* cells were cultured in normal media for 24 h and glucose uptake was determined by performing [^3^H]-2DG uptake assay. Bars represent counts normalized with cell number and plotted relative to control. **B.** Lactate release into the culture medium of Colo357-shScr, Colo357-sh*MUC16*, Capan1-shScr and Capan1-sh*MUC16* cells was determined by performing colorimetric assays. Values were normalized with total cell number and represented relative to control. **C.** Total RNA was isolated from Colo357-shScr, Colo357-sh*MUC16*, Capan1-shScr, and Capan1-sh*MUC16* cells and relative mRNA levels of different genes were quantified by performing real-time PCR. *ACTB* levels were utilized as internal controls. **D.** Protein levels of GLUT1, HKII and LDHA were determined by performing western blotting using Colo357-shScr, Colo357-sh*MUC16*, Capan1-shScr and Capan1-sh*MUC16* cells lysates. β-Tubulin was utilized as an internal control. Values presented are mean ± SEM. *p < 0.05

Because growth and invasive properties of most cancer cells significantly depend on their glycolytic capacity [22], we investigated the effect of *MUC16* knockdown on glucose uptake of pancreatic cancer cells. To study the role of MUC16 on different metabolic properties of pancreatic cancer cells, we established Capan1-Scr, Capan1-sh*MUC16*, Colo357-Scr and Colo357-sh*MUC16* cells. We observed significant reduction in glucose uptake capacity of Colo357-sh*MUC16* and Capan1-sh*MUC16* cells in comparison to scrambled control cells (Figure 1A). As a result of enhanced aerobic glycolysis, cancer cells exhibit enhanced lactate secretion, so we further evaluated the effect of *MUC16* knockdown on lactate secretion. We observed a significant decrease in lactate secretion after *MUC16* knockdown (Figure 1B). Since we observed marked decrease in glucose uptake and lactate secretion after *MUC*16 knockdown, we further evaluated the effect of *MUC16* knockdown on mRNA expression levels of *GLUT1, HKII*, and *LDHA* by performing real-time PCR analysis. We observed significant reduction in *GLUT1* and *HKII* expression after *MUC16* knockdown but no effect on *LDHA* expression (Figure 1C). We also evaluated the effect of *MUC16* knockdown on protein levels of GLUT1, HKII and LDHA and observed decreased expression of GLUT1 and HKII in *MUC16* knockdown cells (Figure 1D). MUC16 protein level is shown in supplementary figure 1 (Figure S1). Overall, our results demonstrate that MUC16 enhances glycolytic gene expression and the glycolytic property of pancreatic cancer cells.

### *MUC16* knockdown pancreatic cancer cells exhibit reduced motility and invasion

**Figure 2:**
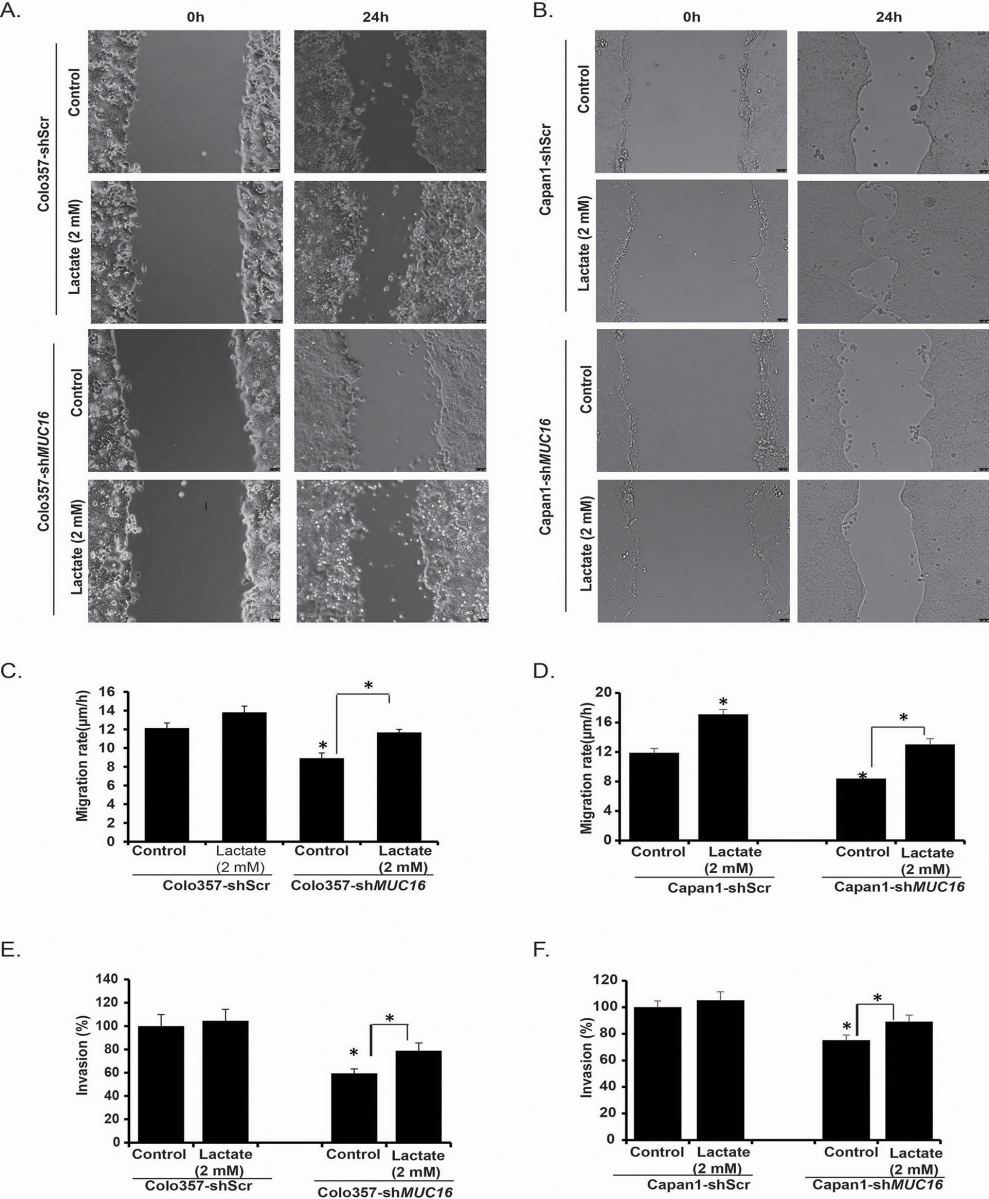
*MUC16* knockdown results in reduced motility/invasion that can be reversed by lactate supplementation **A.** Colo357-shScr, Colo357-sh*MUC16* and **B.** Capan1-shScr, Capan1-sh*MUC16* cells motility was evaluated by scratch wound healing assay for 24 h in the presence or absence of cell culture media supplementation with lactate. Representative bright field images are presented. Bar diagrams represent migration rates of Colo357-shScr and Colo357-sh*MUC16* **C**. and Capan1-shScr and Capan1-sh*MUC16* **D.** with and without lactate supplementation. Invasion potential of Colo357-shScr and Colo357-sh*MUC16* cells **E.** and Capan1-shScr and Capan1-sh*MUC16* cells **F.** was evaluated by matrigel invasion assays for 24 h in the presence or absence of lactate supplementation. Bar diagrams represent percent invading cells in comparison to the controls. Values presented are mean ± SEM. *p < 0.05

It has been shown recently that high glucose levels and increased lactate levels in extracellular milieu promote motility of cancer cells [23]. As we observed reduced glucose uptake by *MUC16* knockdown cells, we further analyzed the role of *MUC16* in cell motility and invasion. We investigated migration properties by performing wound-healing assays. We observed a significant decrease in the rate of migration of Colo357-sh*MUC16* and Capan1-sh*MUC16* cells in comparison to the control cells (Figure 2A–2D). Since *MUC16* knockdown cells also demonstrate decreased secretion of lactate, which is known to regulate tumor cell motility, we next studied if supplementation of culture media with lactate could restore cell migration in *MUC16* knockdown cells. We observed increased cell migration after addition of lactate (Figure 2A–2D). Furthermore, we investigated invasive potential of *MUC16* knockdown cells by performing matrigel invasion assays. We observed significant decrease in invasive properties of Colo357-sh*MUC16* and Capan1-sh*MUC16* cells in comparison to control cells. Similar to migration, we also observed increased cell invasion after addition of lactate to culture media (Figure 2E–2F). Overall, we observed significant inhibition of motility and invasion in *MUC16* knockdown cells in comparison to controls and the inhibition could be reverted by increasing lactate levels in culture media.

### *MUC16* knockdown leads to decreased Akt and mTOR activity

**Figure 3:**
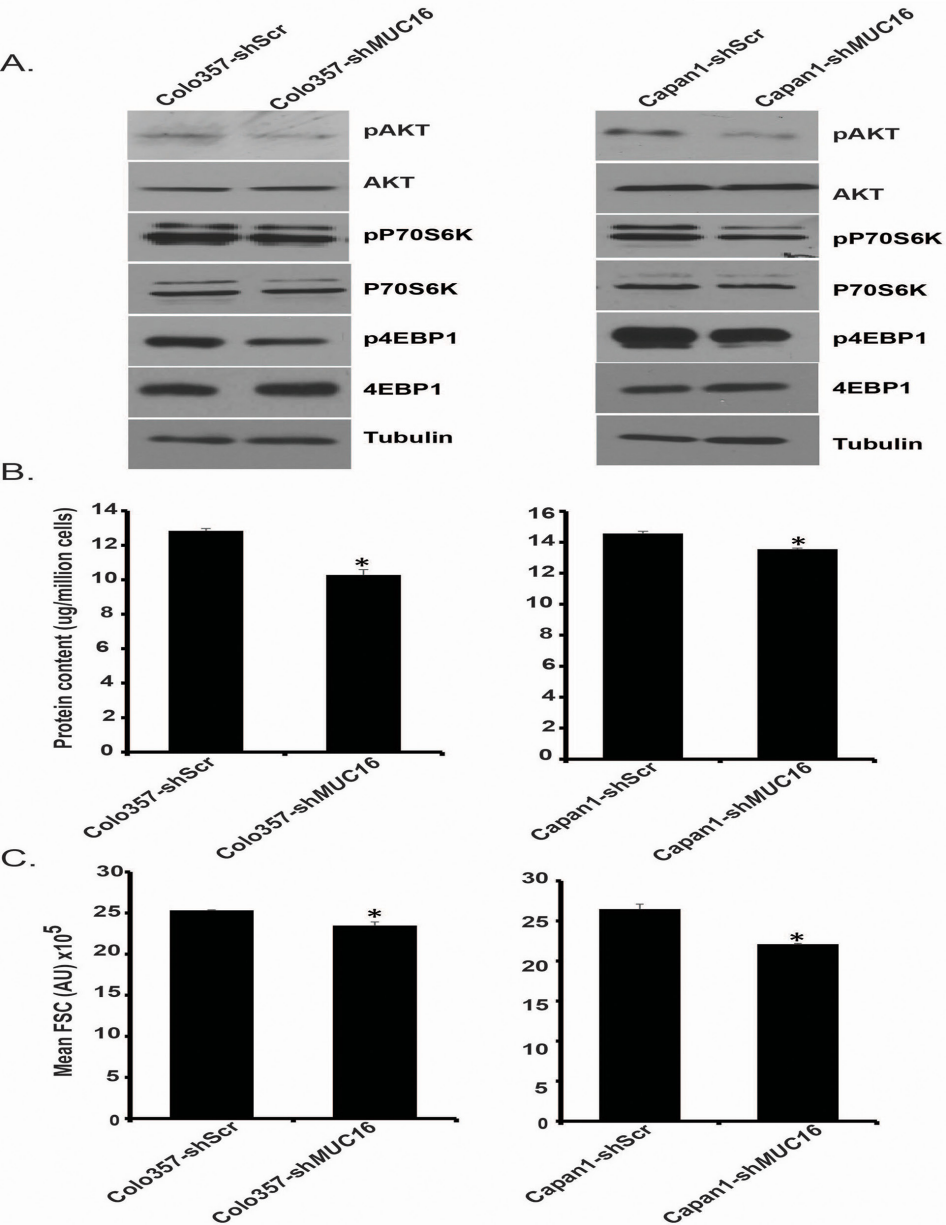
*MUC16* knockdown reduces Akt and mTORC1 activation **A.** Protein expression levels of Akt, pAkt, p70S6K, pP70S6K, 4EBP1, p4EBP1 were determined by western blotting, utilizing Colo357-shScr, Colo357-sh*MUC16*, Capan1-shScr, and Capan1-sh*MUC16* whole cell lysates. β-Tubulin was utilized as an internal control. **B.** Total protein content of Colo357-shScr, Colo357-sh*MUC16*, Capan1-shScr and Capan1-sh*MUC16* cells was evaluated by measuring total protein concentration with Bradford assay and was normalized to the respective cell counts. **C.** Relative cell sizes of Colo357-shScr, Colo357-sh*MUC16*, Capan1-shScr, and Capan1-sh*MUC16* cells were evaluated by utilizing flow cytometry. Mean FSC (forward scatter) values are represented in the bar diagrams. Values presented are mean ± SEM. *p < 0.05

Since we observed alterations in glucose uptake and lactate secretion in *MUC16* knockdown cells, and a corresponding decrease in metabolic gene expression, we next evaluated if these changes were due to decreased signaling activation of upstream regulatory pathways upon *MUC16* knockdown. Hence, we evaluated the effect of *MUC16* knockdown on PI3K-Akt-mTORC1 pathway, which plays an important role in the regulation of cellular metabolism including glycolysis and plays a very significant role in the progression of several types of cancer [24, 25]. We observed a decreased phosphorylation of Akt and mTORC1 target proteins p70S6K and 4EBP1 in Colo357-sh*MUC16* and Capan1-sh*MUC16* cells in comparison to control cells (Figure 3A). The mTOR pathway has emerged as a critical regulator of cellular metabolism which coordinates several anabolic processes leading to increased biosynthetic capacity of the cells [26]. It has been shown that mTORC1 activity promotes the protein synthesis capacity of cells [27]. Hence, we analyzed the relative protein content per cell by measuring total protein content and normalizing it to the cell number. As expected, we observed reduced protein content in *MUC16* knockdown cells (Figure 3B). Recently, it has been shown that metabolic activity of a cell and protein synthesis correlates with cell size [28], so we further evaluated the effect of *MUC16* knockdown on cell size by measuring relative forward side scatter (FSC) mean intensity using flow cytometry. We observed significant decrease in cell size after *MUC16* knockdown (Figure 3C). Overall, these results suggest that *MUC16* knockdown in pancreatic cancer cells leads to decreased Akt and mTORC1 activation and reduced protein synthesis and cell size.

### *MUC16* knockdown leads to decreased c-MYC expression

**Figure 4:**
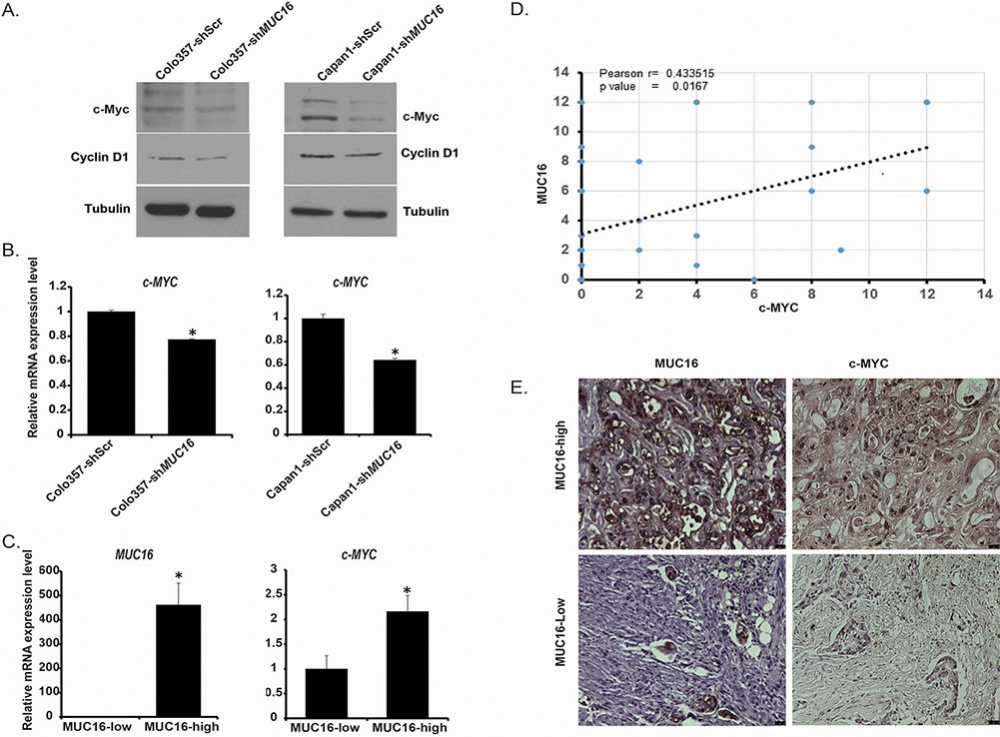
*MUC16* knockdown results in reduced c-Myc expression **A.** c-MYC and Cyclin-D1 expression was determined by western blotting using Colo357-shScr, Colo357-sh*MUC16*, Capan1-shScr and Capan1-sh*MUC16* cells whole cell lysate. β-Tubulin was utilized as an internal control. **B.** Relative c-*MYC* expression in Colo357-shScr, Colo357-sh*MUC16*, Capan1-shScr, and Capan1-sh*MUC16* cells was determined by using qRT-PCR. β-Actin was utilized as an internal control. **C.** Relative c-*MYC* expression in *MUC16-*low and *MUC16* -high patient tumor samples. β-Actin was utilized as an internal control. **D.** Coexpression analysis of MUC16 and c-MYC in human pancreatic cancer samples (*n* = 30). Plotted data are composite immunohistochemistry scores of MUC16 and c-MYC. **E.** Representative immunohistochemistry images of MUC16 and c-MYC in MUC16-high and MUC16-low patient tumors. The size bars at the bottom right corner indicate 25 micron. Values presented are mean ± SEM. *p < 0.05

Proto-oncogene c-MYC is a critical regulator of cellular metabolism and is frequently overexpressed in several types of cancer by different molecular mechanisms [29]. It increases the expression of key glycolytic genes and activates glycolysis in several types of cancer [30]. Furthermore, deregulation of c-MYC expression has been shown in pancreatic cancer [31]. Our results demonstrate down-regulation of glycolytic genes as well as reduced activation of Akt and mTORC1, which can regulate c-MYC expression [32, 33], in *MUC16* knockdown pancreatic cancer cells. Hence, we analyzed c-MYC expression under conditions of *MUC16* knockdown and control. We observed reduced c-MYC expression at protein level (Figure 4A) as well as mRNA level (Figure 4B) in Colo357-sh*MUC16* and Capan1-sh*MUC16* cells in comparison to control cells. We also analyzed protein expression levels of Cyclin D1, which coordinates with c-MYC in tumor initiation and progression [34]. Our results demonstrate reduced Cyclin D1 levels in Colo357-sh*MUC16* and Capan1-sh*MUC16* cells in comparison to control cells (Figure 4A). Furthermore, we analyzed the mRNA level of *c-MYC* in *MUC16*-high and *MUC16*-low human pancreatic cancer tissue specimens. MUC16-high and MUC16-low samples were classified on the basis of staining of MUC16 by immunohistochemistry. We observed significant increase in *c-MYC* expression in MUC16-positive tumor samples (Figure 4C). We then determined c-MYC status in the pancreatic cancer tissue specimens by immunohistochemistry and evaluated if MUC16 levels correlate with c-MYC levels. We observed a positive correlation between MUC16 and c-MYC expression (Figure 4D). Representative immunohistochemistry images of MUC16 and c-MYC are presented in Figure 4E. Overall, our results demonstrate that MUC16 expression regulates the expression of c-MYC, an important metabolic regulator.

### Ectopic expression of c-MYC compensates for the physiological and molecular alterations caused by *MUC16* knockdown

**Figure 5:**
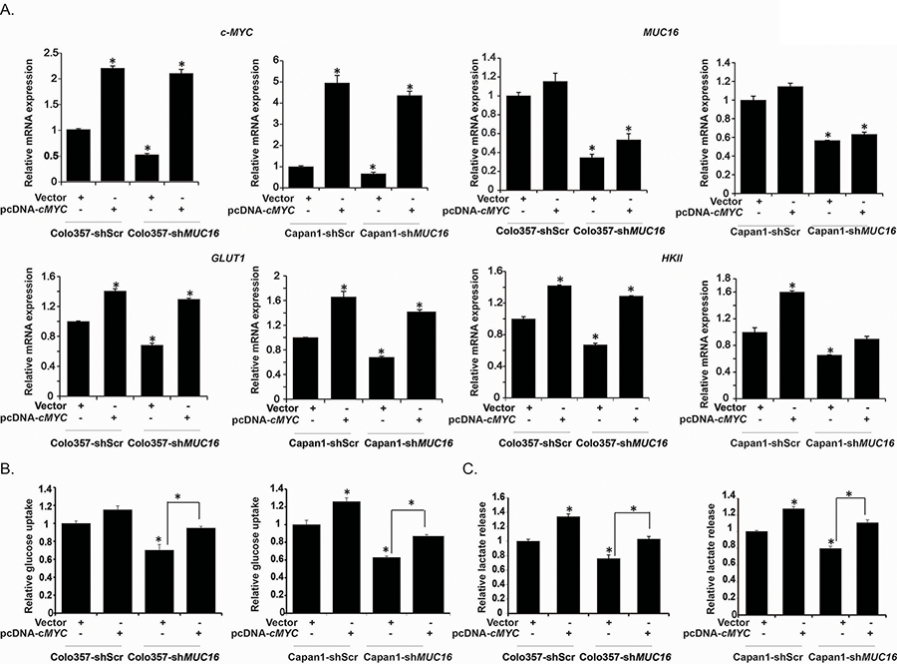
Overexpression of *c-MYC* in *MUC16* knockdown cells reverses the glycolytic inhibition **A.** Colo357-shScr, Colo357-sh*MUC16*, Capan1-shScr and Capan1-sh*MUC16* cells were transiently transfected with vector control or pcDNA3-*cMYC.* After 48 h of transfection total RNA was extracted and relative mRNA expression of indicated genes was quantified by performing qRT-PCR. **B.** Cells transiently transfected with vector control or *c-MYC* were subjected to [^3^H]-2DG uptake assays. Bars represent counts normalized to cell number and plotted relative to that of the vector controls. **C.** Lactate release was determined by colorimetric assay utilizing culture medium of vector control and pcDNA3-*cMYC* transfected Colo357-shScr, Colo357-sh*MUC16*, Capan1-shScr, and Capan1-sh*MUC16* cells. Values are normalized to total cell number and represented relative to that of the controls. Values shown are mean ± SEM. *p < 0.05

Based on our observations indicating reduced levels of c-MYC and reduced expression of metabolic genes downstream of c-MYC in *MUC16* knockdown cells, we next investigated the role of c-MYC expression in *MUC16-*mediated physiological and metabolic alterations. We ectopically expressed *c-MYC* in Colo357-shScr, Colo357-sh*MUC16*, Capan1-shScr and Capan1-sh*MUC16* by transient transfection of pcDNA3-*c*-*MYC* plasmid or vector control and investigated these cells for glucose uptake, lactate secretion and glycolytic gene expression. We observed increased expression of *GLUT1* and *HKII* at mRNA level upon ectopic expression of *c-MYC* (Figure 5A). The increases in mRNA levels of *GLUT1* and *HKII* in *MUC16* knockdown cells were comparable to that of the control cells (Figure 5A). We observed significant increase in glucose uptake (Figure 5B), lactate secretion (Figure 5C) upon ectopic expression of *c-MYC*. Furthermore, *c-MYC* overexpression rescued glucose uptake and lactate secretion in *MUC16* knockdown cells. Overall, these results demonstrate that c-MYC plays an important role in MUC16-mediated metabolic alterations.

### MUC16 expression regulates glycolytic and nucleotide metabolism in cultured cells and human pancreatic tumors

**Figure 6:**
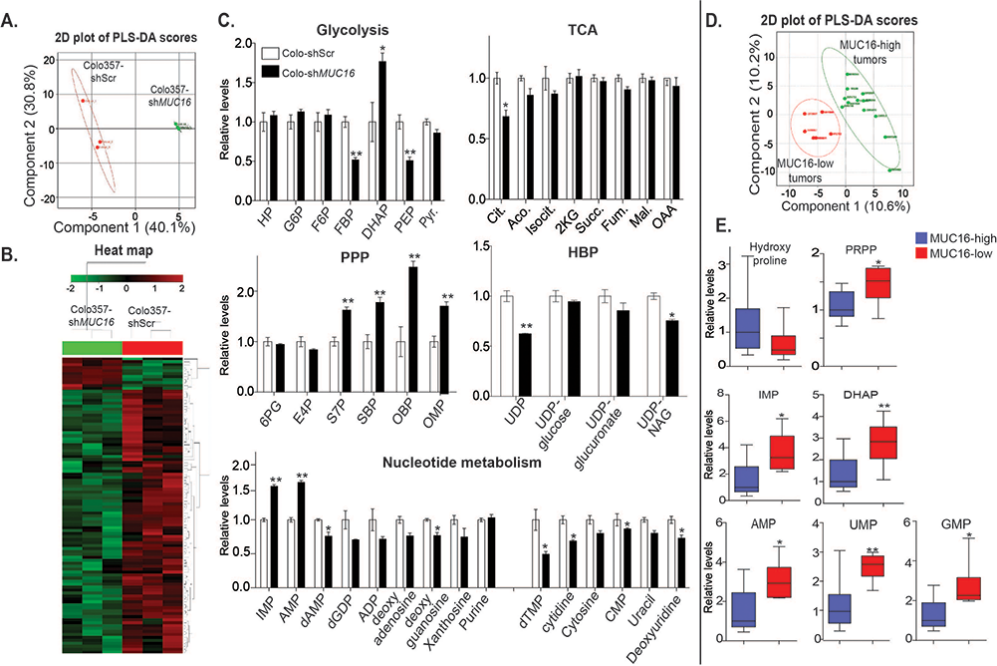
MUC16 alters glycolysis, PPP and nucleotide metabolism in both pancreatic cancer cells and MUC16 expressing tumors Polar metabolites extracted from Colo357 cells (shScr and sh*MUC16*, 3 replicates each and normalized to protein content) and human pancreatic tumors (MUC16-high and MUC16-low) were analyzed by directed metabolomics-MRM method using LC-MS/MS (Schimadzu HPLC-AB SCIEX QTRAP5500) platform. LC-MS/MS Data was analyzed utilizing Analyst and Metaboanalyst softwares. **A.** PLS-DA (partial least squares discriminant analysis) plot generated from LC-MS/MS data of Colo357-shScr & Colo357-sh*MUC16* cell metabolites. **B.** Heat map of metabolites generated from LC-MS/MS data of Colo357-shScr & Colo357-sh*MUC16* cells. **C.** Relative metabolites level in Colo357-shScr & Colo357-sh*MUC16* cells determined by LC-MS/MS. **D.** PLS-DA plot generated from LC-MS/MS data of MUC16-high (*n* = 12) and MUC16-low (*n* = 6) pancreatic tumor specimen metabolites. **E.** Relative metabolites level in MUC16-high (*n* = 12) and MUC16-low (*n* = 6) pancreatic tumor specimen determined by LC-MS/MS. (TCA, PPP and HBP indicate-TCA cycle, pentose phosphate pathway and hexosamine biosynthesis pathways, respectively. * and **-indicate *p* < 0.05 and *p* < 0.01, respectively).

In order to identify the possible role of MUC16 in modulating distinct metabolic pathways in tumor cells, we evaluated the polar metabolite contents of control and *MUC16* knockdown Colo357 pancreatic cancer cells and that of the MUC16-high and MUC16-low rapid autopsy pancreatic tumor specimens, as determined by IHC. Based on the overall metabolite profiles, control and *MUC16* knockdown cultured cells, as well as MUC16-high and MUC16-low human pancreatic tumors, segregated in distinct clusters, in a MUC16 expression-dependent manner, in the respective 2D-PLS-DA (partial least squares discriminant analysis) plots (Figure 6A and 6D). Furthermore, MUC16-expressing cells clustered together based on their individual metabolite differences, as evident from the unsupervised hierarchical clustering in heat maps (Figure 6B). Corroborating our findings on increased glucose uptake under *in vitro* conditions, we observed higher levels of phosphoenolpyruvate, a glycolytic intermediate downstream of dihyroxyacetone phosphate (DHAP), in MUC16-expressing cells, as evident from the LC-MS/MS analyses (Figure 6C). Higher DHAP levels, which indicate lower glycolytic flux, were observed in *MUC16* knockdown cells as well as MUC16-low tumors (Figure 6E). However, MUC16 expression did not alter most of the metabolite levels in the tricarboxylic acid cycle (TCA) (Figure 6C). Glycolysis can supplement the intermediates of the pentose phosphate pathway (PPP), required for nucleotide biosynthesis in tumor cells. MUC16-expressing cells also exhibited higher nucleotide metabolism intermediates. However, the precursor inosine mono-phosphate (IMP) and adenosine mono-phosphate (AMP) were significantly higher in *MUC16* knockdown cells, indicating the possibility for lower flux downstream of these metabolites under *MUC16* knockdown conditions. Furthermore, IMP and AMP levels were higher in the MUC16-low tumors, similar to that of the *MUC16* knockdown cells (Figure 6E).

## DISCUSSION

Altered metabolism is considered a hallmark of neoplastic cells [35]. Cancer cells adopt an altered metabolic state to fulfill their bioenergetic and biomass requirements for maintaining continuous growth and proliferation [15]. Some metabolic adaptations also provide protection against stress conditions like increased reactive oxygen species (ROS) levels and nutrient deficiency [36]. Oncogenes and tumor suppressors have been reported to play a significant role in metabolic adaptation of pancreatic cancer cells by modulating expression and activation of different metabolic pathway genes [37].

MUC16 expression is upregulated in pancreatic cancer, with strong upregulation in metastatic lesions [12]. Furthermore, MUC16 expression correlates with the recurrence rate in pancreatic cancer [38]. In case of ovarian cancer, MUC16/CA-125 is a well-established biomarker [39], and regulates cancer progression and metastasis [40]. Keeping in view the oncogenic functions of MUC16, we investigated its role in metabolic reprogramming of pancreatic cancer cells. Our results demonstrate that *MUC16* knockdown reduces expression of key glycolytic genes and glycolytic activity of the pancreatic cancer cells. Aberrant glycolysis is reported in several types of cancers and is considered a key metabolic alteration by which cancer cells fulfill their biomass and energy requirements [22]. We observed a reduction in glucose uptake in *MUC16* knockdown cells. Glucose transporters facilitate the entry of glucose into cancer cells. Glucose transporter GLUT1 is overexpressed in multiple human solid tumors and correlates negatively with patient prognosis [41–43]. We observed reduced expression of *GLUT1* mRNA and protein in *MUC16* knockdown cells, which indicates regulation of GLUT1 expression by MUC16. We also investigated the effect of glucose and glutamine deprivation on the survival of *MUC16* knockdown cells and observed that *MUC16* knockdown cells are less sensitive to glucose and glutamine deprivation (Figure S2). Recently, our lab has established that another important mucin family member, MUC1, regulates glucose metabolism in pancreatic cancer [20]. In contrast to MUC1, the effect of MUC16 on glycolysis is mediated by altered expression of a smaller set of glycolytic genes. MUC16 knockdown downregulated the expression of hexokinase II (HK II), a key enzyme of glycolysis that is upregulated in several types of cancers and whose ablation leads to reduced tumor growth [44].

Altered glucose metabolism or aerobic glycolysis generates a tumor friendly niche, especially by creating an acidic tumor microenvironment through increased lactate secretion [16]. We observed reduced lactate secretion in *MUC16* knockdown pancreatic cancer cells. Interestingly, supplementation of cell culture media with lactate led to a recovery of motility and invasiveness of *MUC16* knockdown cells. However, we did not observe a similar magnitude of effect of lactate on motility and invasiveness of control cells, suggesting that lactate levels above a certain threshold may not have any physiological impact on motility and invasiveness. These results indicate that lactate plays an important role in *MUC16-*mediated motility and invasiveness of pancreatic cancer cells. Lactate is considered a key player in development and metastasis of cancer. Lactate also promotes tumor metastasis by induction of hyaluronan synthesis from stromal cells. Furthermore, radioresistance of tumors is also correlated with lactate secretion capacity [45].

The PI3K-Akt-mTOR pathway controls several aspects of cancer cells including, growth, proliferation, cell cycle, motility, genomic instability and metabolism [46]. The mTOR pathway coordinates several signaling pathways and controls the cellular physiology by regulating several metabolic and biosynthetic processes such as protein synthesis and lipogenesis [26]. mTOR activation is also known to enhance lactate production [47]. The mTOR pathway is commonly deregulated in several types of human cancers and its activity is regulated in response to the availability of growth factors and nutrients in the extracellular milieu [48]. We observed reduced phosphorylation of Akt and decreased activity of mTOR in *MUC16* knockdown cells, along with reduced cell size and cellular protein content. This indicates that MUC16 plays an important role in regulating cellular biosynthetic processes through modulation of mTORC1 signaling. Downstream to the mTOR pathway, c-MYC oncogene plays a very important role in the pathogenesis of multiple malignancies. It serves as a critical regulator of cellular metabolism and biosynthetic processes [29]. Recently, it has been shown that c-MYC and mTORC1 converge at a common point to coordinate the process of protein synthesis, thus they regulate cellular growth in a cooperative manner [49]. c-MYC also downregulates the expression of TSC2, a negative regulator of mTOR, to maintain a feed-forward signaling loop [50]. We observed reduced expression of c-MYC in *MUC16* knockdown cells. Furthermore, after ectopic expression of *c-MYC* in *MUC16* knockdown cells, we were able to restore metabolic activity that was compromised due to *MUC16* knockdown. Overall, our result demonstrates that MUC16 mediated *c-MYC* expression regulates physiology and biosynthetic processes of pancreatic cancer cells.

Our mass spectrometery-based metabolomics study of *MUC16* knockdown pancreatic cancer cells indicates that *MUC16* enhances glucose and nucleotide metabolism in pancreatic cancer cells. We observed higher glycolytic intermediates in *MUC16* expressing cells in comparison to the *MUC16* knockdown cells. We observed higher DHAP levels, but reduced PEP levels in *MUC16* knockdown cells, suggesting a potential metabolic blockage in the enzymatic activities that catalyze the glycolytic flux of carbon downstream of DHAP. As we observed reduced glycolytic flux in *MUC16* knockdown cells, we also investigated the effect of dichloroacetate (DCA), which increases flux of the glycolysis end product pyruvate to the TCA cycle, on cell survival [51]. However, we observed no significant effect on cell survival (Figure S3). Since some pentose phosphate pathway intermediates were high in *MUC16* knockdown cells, we further determined the NADPH and ROS level in *MUC16* knockdown cells. We did not observe significant differences in NADPH level (Figure S4A), however MUC16 knockdown cells did exhibit higher ROS levels (Figure S4B). These results indicate that metabolic reprogramming induced by MUC16 plays an important role in ROS homeostasis in pancreatic cancer cells. However, the changes in ROS levels are independent of MUC16-induced flux in pentose phosphate pathway.

Our mass spectrometry-based metabolomics studies provide strong support to our *in vitro* observation that *MUC16* knockdown cells demonstrate reduced glucose uptake. We also observed decreased carbon flux into the nucleotide biosynthesis pathways in *MUC16* knockdown cells. Thus, our studies indicate that *MUC16* activates key bioenergetic and biosynthetic processes by regulating energy metabolism and nucleotide synthesis. Furthermore, our metabolomics studies utilizing human pancreatic tumor specimens also indicate alterations in nucleotide intermediates and differential overall metabolite profiles, in a MUC16 expression-dependent manner. Overall, these findings demonstrate an important role of *MUC16* in metabolic reprogramming in pancreatic cancer.

In conclusion, our findings indicate that MUC16 regulates cellular metabolism. Metabolic reprogramming induced by MUC16 increases the MUC16-mediated cellular invasion and motility. MUC16 knockdown cells exhibit reduced activity of the mTOR pathway and decreased levels of the mTOR Diego downstream target c-MYC, which is a key regulator of cell growth, proliferation and metabolism. Alterations in c-MYC expression seem to be at least in part responsible for MUC16-mediated reprogramming of cellular metabolism. We observed a positive correlation between MUC16 and c-MYC expression in human pancreatic tumor specimens. The MUC16-induced alterations in glycolytic and nucleotide metabolism in culture models overlap with that of the human pancreatic tumor specimens, indicating the broader applicability of our findings to human pancreatic cancer.

## MATERIALS AND METHODS

### Cells and reagents

The human pancreatic cancer cell lines Capan1 and Colo357 were obtained from the American Type Culture Collection. Capan1-shScr, Colo357-shScr, Capan1-sh*MUC16* and Colo357-sh*MUC16* cells were prepared by retrovirus-mediated transduction of scrambled and *MUC16-*specific shRNA constructs. Oligonucleotide sequence of *MUC16* shRNA is provided in Table S1. All the cell lines were cultured in DMEM supplemented with 10% fetal bovine serum (FBS), penicillin (100 mg/mL), and streptomycin (100 mg/ml) and incubated at 37°C in a humidified chamber with 5% CO_2_. pcDNA3-c*MYC* plasmid was purchased from Addgene, Cambridge, MA, USA.

### Glucose uptake assay

Glucose uptake was determined as described previously [52]. Briefly, Capan1-shScr, Colo357-shScr, Capan1-sh*MUC16* and Colo357-sh*MUC16* cells (5×10^4^ cells per well) were seeded in 24-well plates. After overnight culture, cells were washed twice with phosphate buffered saline (PBS; pH 7.4), starved for glucose for 2 h and, then incubated for 20 min with 1 μCi [^3^H]-2-deoxyglucose (DG) for glucose uptake. After incubation, cells were washed with PBS and lysed with 1% SDS. The lysates were then subjected to [^3^H] counting by utilizing a scintillation counter. Scintillation counts from cells treated with labeled and excess unlabeled 2-DG were utilized as controls for base-line correction. The results were normalized to the respective cell counts.

### Lactate secretion assay

Capan1-shScr, Colo357-shScr, Capan1-sh*MUC16* and Colo357-sh*MUC16* cells (5×10^4^ cells per well) were seeded in 24-well plates in phenol-red free DMEM with 10% fetal bovine serum. After overnight incubation at standard culture conditions, culture supernatants were utilized for determining lactate release. The assay was performed by utilizing a Lactate Assay Kit (Eton Bioscience Inc, San Diego, CA, USA), as per the manufacturer's protocol. Lactate amount was normalized to cell counts.

### Gene expression analysis by qRT-PCR

Gene expression analysis using qRT-PCR was performed as described previously [53]. Total RNA was isolated from cells with TRIzol reagent (Invitrogen, Carlsbad, CA, USA), according to the manufacturer's protocol. Total RNA (5 μg) was reverse transcribed by utilizing Verso-cDNA synthesis kit (Thermo-Scientific, Waltham, MA, USA) according to the manufacturer's guidelines. qRT-PCR was performed with gene specific primers at 95°C for 10s, 60°C for 60s (40 cycles) in a 10 μl reaction mix, with an ABI 7500 thermocycler. Reaction mix was prepared for 3 μl cDNA, 2 μl primers and 5 μl SYBR Green master mix (Applied Biosystems, Grand Island, NY, USA), per reaction. Beta-actin was utilized as an internal control. The sequence of primers used in the study is given in Table S2. Quantification was performed with the ΔΔCt method.

### Western blotting

Western blotting of proteins was performed as described previously [20]. Briefly, for western blotting, cells were washed twice with cold PBS and lysed in radioimmuno precipitation assay (RIPA) lysis buffer by incubating at 4°C on a rotatory shaker for 30 min. Cell debris was removed by centrifugation at 13,000 rpm for 10 min and the supernatant was collected. Protein content was measured by Bradford assay. Equal amounts of denatured proteins were separated by electrophoresis using SDS-PAGE gels and transferred to activated PVDF membranes. The membranes were probed with primary antibodies against GLUT1 (Abcam, Cambridge, UK), c-Myc (clone 9E10; Santa Cruz Biotechnology, Dallas, Texas, USA), HKII, pP70S6K, P70S6K, p4EBP1, 4EBP1 (Cell Signaling Technology, Danvers, MA, USA) and beta-tubulin (Clone E7 from Developmental Studies Hybridoma Bank, Iowa City, IA).

### Transient transfection of plasmid

Capan1-shScr, Capan1-sh*MUC16*, Colo357-shScr and Colo357-sh*MUC16* cells were transfected with pcDNA3-c*MYC* and the vector control using X-treme GENE 9 transfection reagent (Roche Diagnostics, Indianapolis, Indiana, USA) as per the manufacturer's protocol.

### Cell migration assay

Cell migration was assessed by performing wound-healing assays. Capan1-shScr, Colo357-shScr, Capan1-sh*MUC16* and Colo357-sh*MUC16* cells (2×10^6^ cells per well) were seeded in 12-well plates and cultured until they became confluent. A scratch was made using a 200 μl pipette tip and bright field images were captured at zero time point and after 24 h using inverted microscope (Leica, DMI600B) and images were processed using Leica LAS AF software. Migration rate was calculated by dividing the distance travelled by the cells with time.

### Cell invasion assay

Cell invasion assay was performed as described previously [54]. Briefly, Capan1-shScr, Colo357-shScr, Capan1-sh*MUC16* and Colo357-sh*MUC16* cells were seeded in serum-free medium on 24-well transwell inserts (8 μm pore size), which were pre-coated with matrigel (BD BioCoat Matrigel Invasion Chamber, BD Bioscience, San Jose, CA, USA). The transwell inserts were incubated for 24 h with bottom chamber containing 10% FBS. After completion of incubation, upper surface cells were removed by cotton-tipped swab and lower membranes were isolated with a pair of forceps and a fine scalpel. The membranes were stained by utilizing Diff-Quik stain (Polyscience Inc., Warington, PA, USA), as per the manufacturer's protocol. After staining, the membranes were mounted on glass slides and covered with a cover slip. Invaded cells were counted in ten fields by utilizing an inverted microscope (Leica, DMI600B) and the percent invasion was calculated relative to the control cells.

### Immunohistochemistry

Immunohistochemistry was performed as described previously [19]. Normal and pancreatic cancer tissue arrays were obtained from rapid autopsy program of UNMC, Omaha, NE, USA. MUC16 mouse monoclonal antibody (M11 clone, manufactured by Dako, Carpinteria, CA, USA) and c-Myc (9E10) antibody (Santa Cruz Biotechnology, CA, USA) were utilized as primary antibodies.

### Metabolite extraction and MS sample preparation

Metabolite extraction was performed as described earlier [52], with some modifications. After confirming 80% confluence of the cells, we replaced the media with fresh media for 2 h before metabolite extraction. Media was aspirated and the cells were washed twice with PBS to remove remnants of the media before lysing the cells. The polar metabolites were then extracted with cryogenically cold 80% methanol/water mixture. LC-MS grade water (Sigma, St Louis, MO, USA) and LC-MS grade methanol (Fisher Scientific, Pittsburgh, PA, USA) were utilized. The cells from the cold plates were scraped with a cell scraper, pipetted into an Eppendorf tube, and centrifuged at 13000 rpm for 5 min to collect the supernatant. The samples were dried using a speed vacuum evaporator (Savant Speed Vac^®^ Plus, Thermo Electron Corporation, USA) to evaporate the methanol and lyophilized by using freeze dry system (Labconco, Kansas City, USA) to remove the water consecutively. The dried sample was made ready for mass spectrometry by dissolving in LC-MS grade water. For extraction of metabolites from tumor tissues, frozen tumor samples were weighed and homogenized using a hand held homogenizer. Homogenized tumor samples were collected and cold 80% methanol was added to these samples maintaining similar proportion of 80% methanol to the tumor weights. Samples were vortexed and maintained at −80°C for 10 min, then briefly sonicated for 3 pulses on dry ice, followed by centrifugation at 3400 rpm at 4°C for 10 min. Supernatants were collected, concentrated and subjected to liquid chromatography/tandem mass spectrometry (LC-MS/MS) as described below.

### LC-MS/MS experiment and analysis

Lyophilized concentrates were resuspended in equal volumes of LC-MS grade water and subjected to LC-MS/MS analysis, using a multiple reaction monitoring (MRM) method by utilizing AB SCIEX 5500 QTRAP, as described previously [55]. Data acquisition was carried out using Analyst™1.6 software (AB SCIEX) and peaks were integrated with Multiquant™ (AB SCIEX). Peak areas were normalized with the respective protein concentrations and the resultant peak areas were subjected to relative quantification analyses with MetaboAnalyst 2.0 [56].

### Patient specimens

PDAC samples from 30 patients with metastatic PDAC from the University of Nebraska Medical Center Rapid Autopsy Pancreatic Program (RAP) were evaluated for differentially expressed genes (at protein levels) and metabolites. Human pancreatic tumors, and uninvolved specimens harvested by rapid autopsy from preconsented decedents previously diagnosed with pancreatic ductal carcinoma, were obtained from the University of Nebraska Medical Center's Tissue Bank through the Rapid Autopsy Pancreatic program. Written consent was obtained from patients prior to death. The Rapid Autopsy sample collection was performed as per the University of Nebraska Medical Center Institutional Review Board (UNMC-IRB) approval (approval # 091-01-FB).

### Statistical analysis

Comparisons between two groups were performed utilizing Student's *t*-test and comparison for the response of treatments was performed with ANOVA (one-way; graph Pad Prism version 4.03). Tukey's Post-hoc analysis was utilized for pair-wise comparisons.

## SUPPLEMENTARY FIGURES AND TABLES


