# Supplementary material for: MUC16-mediated activation of mTOR and c-MYC reprograms pancreatic cancer metabolism
Source: Oncotarget. 2015 Jun 3;6(22):19118–31. doi: 10.18632/oncotarget.4078 (PMC4662479; doi:10.18632/oncotarget.4078)
Supplement: Supplementary file 1 [file oncotarget-06-19118-s001.pdf]

SUPPLEMENTARY FIGURES AND TABLES

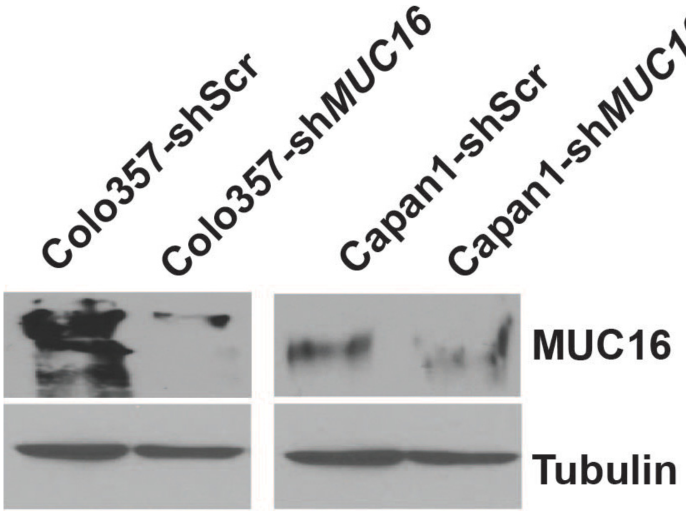

Supplementary Figure S1: *MUC16* knockdown in Colo357 and Capan1 cells. Immunoblots of MUC16 in Colo357-shScr, Colo357-sh*MUC16*, Capan1-shScr and Capan1-sh*MUC16*.

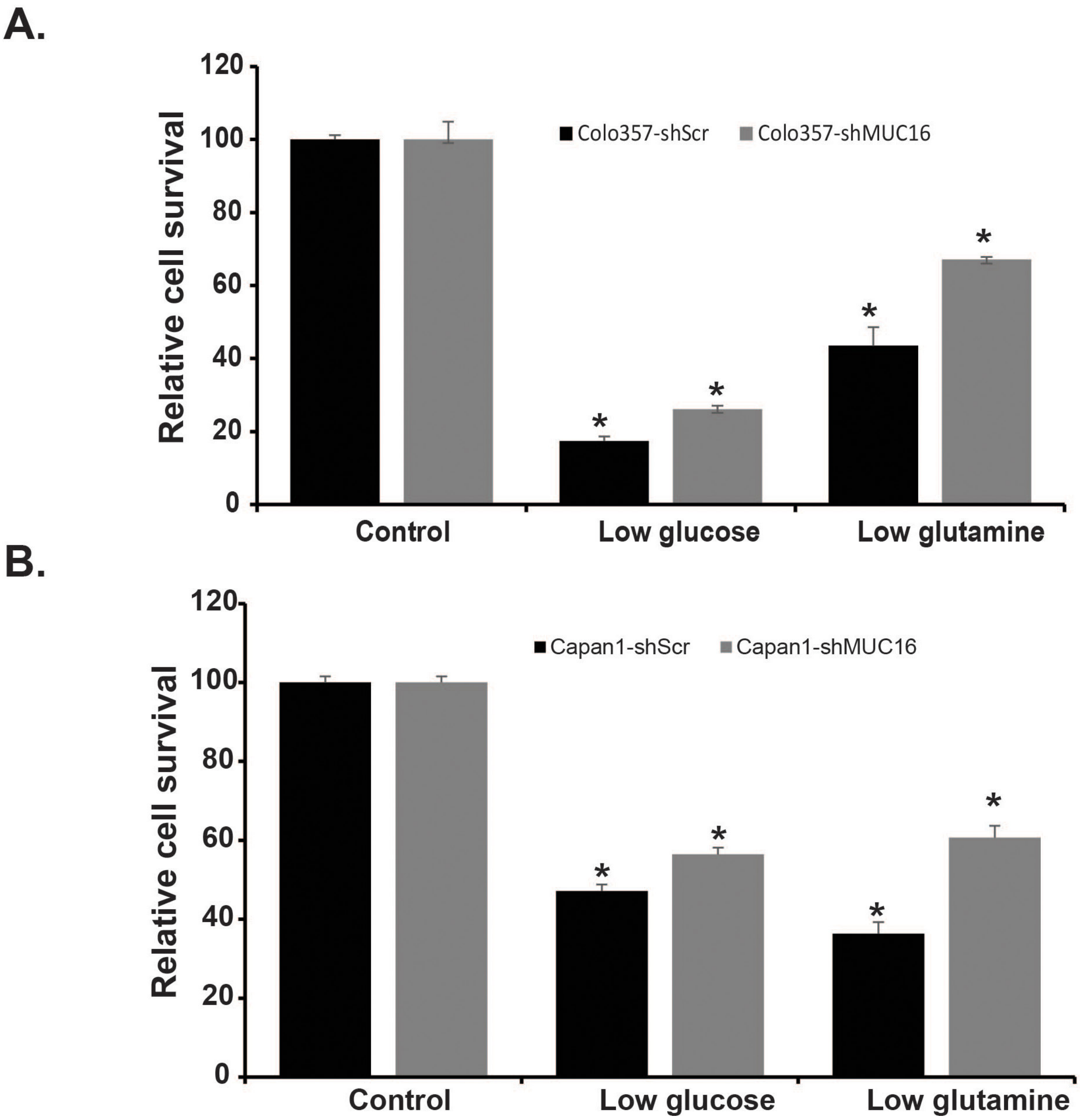

Supplementary Figure S2: Effect of low glucose and low glutamine on *MUC16* knockdown and control cells. A. Colo357-shScr and Colo357-sh*MUC16* **B.** Capan1-shScr and Capan1-sh*MUC16* cells were cultured under control, low glucose or glutamine conditions. After 72 hours, cell survival was determined by MTT assay. Bar diagrams represent percent cell survival in comparison to the controls. Values presented are mean ± SEM. *$p < 0.05$

A.

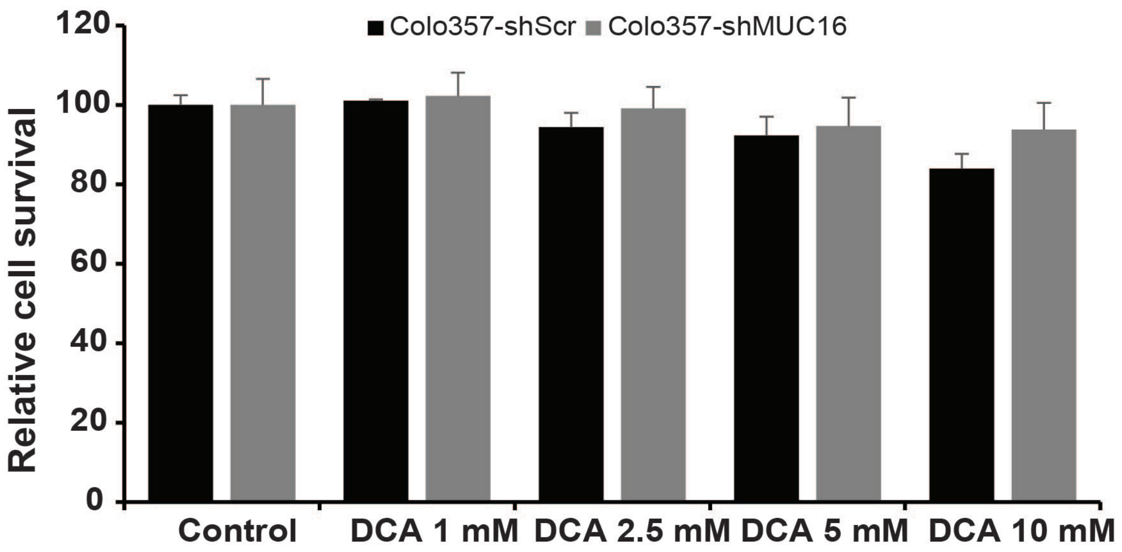

B.

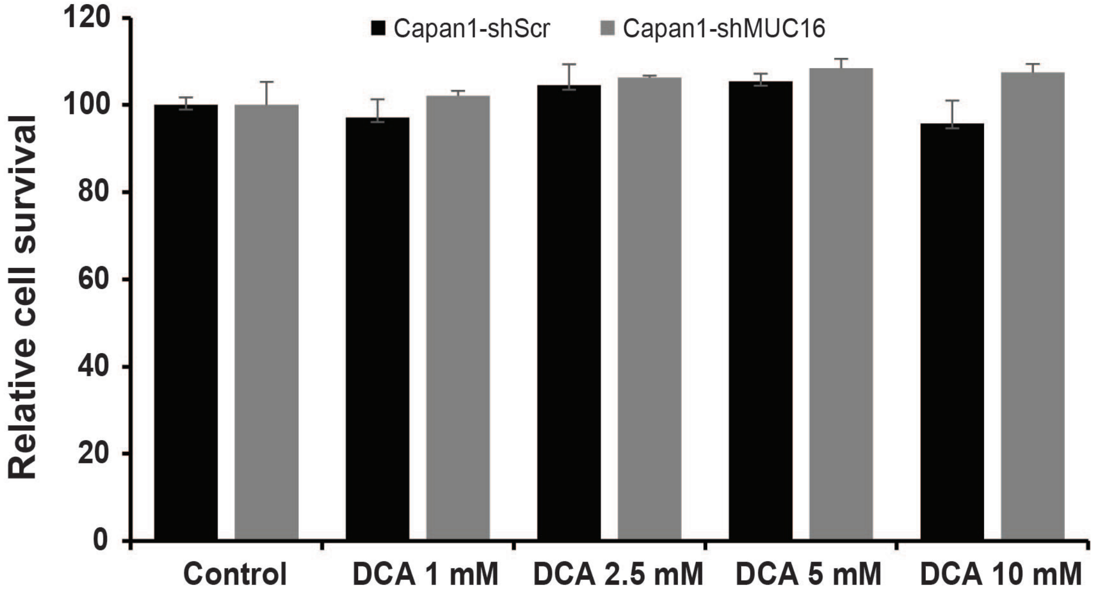

Supplementary Figure S3: Effect of DCA on *MUC16* knockdown and control cells. A. Colo357-shScr and Colo357-sh*MUC16* **B.** Capan1-shScr and Capan1-sh*MUC16* cells were treated with different dose of DCA. After 72 hours, cell survival was determined by MTT assay. Bar diagrams represent percent cell survival in comparison to the controls. Values presented are mean ± SEM.

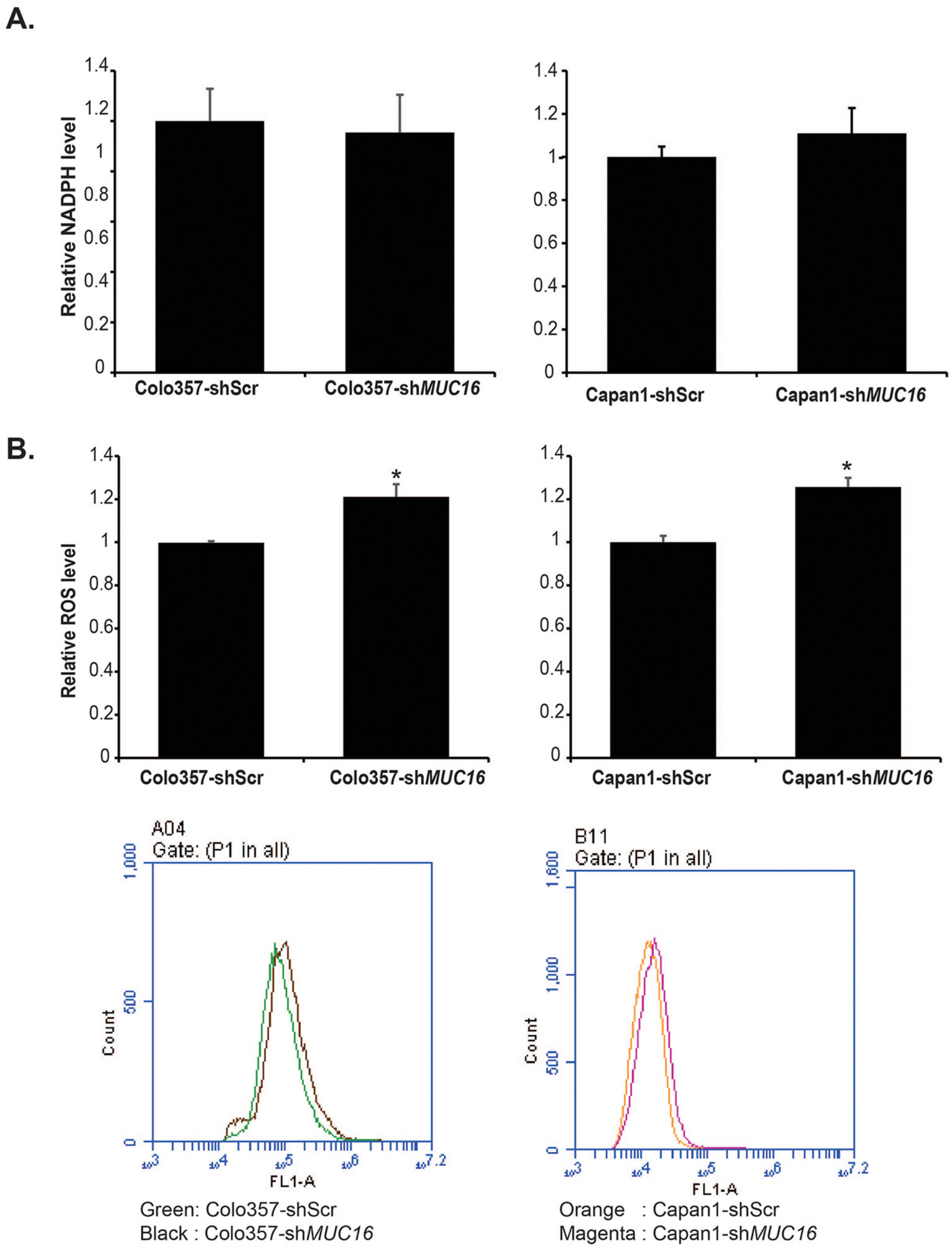

Supplementary Figure S4: Effect of *MUC16* knockdown on cellular NADPH and ROS levels. A. Relative NADPH level in Colo357-shScr, Colo357-sh*MUC16*, Capan1-shScr and Capan1-sh*MUC16* cells measured by a colorimetric kit (BioVision, CA, USA). **B.** Relative ROS level in Colo357-shScr, Colo357-sh*MUC16*, Capan1-shScr and Capan1-sh*MUC16* cells measured by using Carboxy-DCFDA (5-(and-6)-Carboxy-2′,7′-Dichlorofluorescein Diacetate).*$p < 0.05$.

Supplementary Table S1: sh*MUC16* sequence

Sense, 5′-CTGCATGTACTCCCATCTCTTCAAGAGAGAGATGGGAGTAGATGCAG-3′,
Antisense, 5′-CTGCATCTACTCCCATCTCTCTCTTGAAGAGATGGGAGTAGATGCAG-3′

Supplementary Table S2: Primer sequences

Gene	Sequence (5′-3′)
GLUT1-F	GATTGGCTCCTTCTCTGTGG
GLUT1-R	TCAAAGGACTTGCCCAGTTT
HKII-F	GAGCCACCACTCACCCTACT
HKII-R	ACCCAAAGCACACACGGAAGT
LDHA-F	TTGACCTACGTGGCTTGGAAG
LDHA-R	GGTAACGGAATCGGGCTGAAT
cMYC-F	GGACGACGACGAGACCTTCATCAA
cMYC-R	CCAGCTTCTCTGAGACGAGCTT
MUC16-F	ACATCAACTCCTGCCTTCCCAGAA
MUC16-R	ACCAGTGGGCATTCCAGAAAGAGA
Actin-F	GTCCACCGCAAATGCTTCTA
Actin-R	TGCTGTCACCTTCACCGTTC
